# Direct Homogeneous Synthesis of Compounds with Two O Atoms and Long-Chain Hydrocarbons from CO and H_2_: Co–Ru/N-methylpyrrolidone Catalyst

**DOI:** 10.3390/molecules28176341

**Published:** 2023-08-30

**Authors:** Anton Lvovich Maximov, Mayya V. Kulikova, Alexey E. Kuz’min, Mikhail I. Ivantsov

**Affiliations:** A. V. Topchiev Institute of Petrochemical Synthesis, Russian Academy of Sciences, Leninsky Prospekt 29, Moscow 119991, Russia; max@ips.ac.ru (A.L.M.); ivantsov@ips.ac.ru (M.I.I.)

**Keywords:** homogeneous catalysis, synthesis gas, N-methylpyrrolidone, acetic acid, acetates, long-chain hydrocarbons, Anderson-Schultz-Flory distribution

## Abstract

The homogeneous acetic acid synthesis-type Ru–Co–Li/N-methylpyrrolidone catalyst for CO and H_2_ transformations has been studied at moderately high pressures. For 1CO:2H_2_, low acetic acid selectivity has been observed, along with remarkable methyl acetate selectivity, the absence of aldehydes and ethyl acetate and sharp deviations from the Anderson-Schultz-Flory distribution for both alcaohols and long-chain hydrocarbons. For 1CO:1H_2_ and slightly elevated pressure, acetic acid selectivity slightly increased, notable ethyl acetate formation was detected, and both long-chain hydrocarbons and alcohols disappeared. Hypotheses are discussed about the direct parallel formation of all observed product groups (hydrocarbons, alcohols, esters, and acetic acid) and hydrocarbon chain growth limitations according to the formed Ru–Co cluster size in the presence of the aforementioned catalytic system.

## 1. Introduction

The conversion of carbon oxides and hydrogen in the presence of homogeneous catalysts represents a broad, although not always evenly investigated and implemented, area of catalysis. On the one hand, if some third component (methanol or an alkene) is used, one can see well-known processes such as carbonylation and hydroformylation [1,2,3].

For the carbonylation reaction, carbonyls of Ni, Co, Pd, Ru, and Rh have been used as catalysts since the 1930s (W. Reppe [4]). Phosphine-substituted metal carbonyls are especially active and reveal good solubility in the reaction media. The promoters of carbonylation catalysts include Se, Cu, Zn, and nitrogen bases (pyridine, in particular). Perhaps the most famous carbonylation process is the process of acetic acid production from methanol; its worldwide production volume exceeds 2 million t/y. According to the BASF, the synthesis is carried out in the presence of CoI_2_ or Co(OAc)_2_ and is promoted by iodine or its compounds. The synthesis proceeds at 200–250 °C and 50–70 MPa carbon monoxide pressure. According to Monsanto, the synthesis is conducted in the presence of a modified Rh catalyst with additives of iodine or its compounds under milder conditions—temperature of 150–200 °C; pressure of CO 3.0–6.0 MPa [4].

Hydroformylation (the industrial implementation of this process is often referred to as “oxo-synthesis”) was discovered by Roelen in 1938. The most interesting is the hydroformylation of olefins, which occurs in the presence of catalysts—carbonyl complexes of transition metals (mainly Co or Rh). The resulting aldehydes can be converted into the corresponding primary alcohols by hydrogenation on heterogeneous catalysts [5,6]. Such “oxo-alcohols” are of the greatest importance compared to other reaction products: hydroformylation is the only industrial process capable of producing long-chain aliphatic alcohols.

On the other hand, direct transformations of synthesis gasses using homogeneous catalysis are not yet as well established as carbonylation and hydroformylation processes or the “classical” heterogeneous Fischer-Tropsch synthesis. Since the 1970s, many original works and several reviews have been devoted to their study [7,8,9,10,11,12], adequately illustrating the most important problem in this field: the significant difficulties of conducting synthesis gas conversion on the same catalytic system under the same conditions include the following:

(a) For CO reduction, two-component carbonyl-late transition metal (Re, Fe, cluster, or polymetallic) hydride systems are required since single-centered hydride carbonylation is thermodynamically unfavorable.
(b) For the effective formation of the C–C bond in homogeneous complexes, it is usually necessary to have Lewis-type promoters with optimal acidity. Polymetallic hydrides with a possible content of Ta, Ln, Y, and Ti promote chain growth without (ethenediolates) or with partial C–O breaking (allyl oxides, squarats, etc.) [12].
(c) Long-chain product elimination requires the presence of sufficiently strong electrophiles and leads to the problem of the combination of protonic and hydride hydrogen forms at the same conditions. Therefore, it becomes necessary to use very high pressure and elevated temperatures.

It was under such conditions in the 1970–1980s that a one-stage selective conversion of CO and H_2_ into acetic acid was carried out [13]. Union Carbide’s investigations [14,15,16] led to a 79% acetic acid selectivity (acetaldehyde 10, methane 10, and ethanol approximately 1%) at 250–350 °C and up to 35 MPa in the presence of Rh/SiO_2_. In the presence of Ir/SiO_2_, selectivity for acetic acid could reach 82% at a price of multiple decreases in the overall productivity compared with Rh/SiO_2_. The main problem of this process is an acid selectivity decrease with time. However, it was later found that, with different catalyst support (NaY zeolite) or synthesis conditions (reduced pressure, for example), process stability could improve at the cost of a significant (2–3 times) decrease in the initial acid selectivity [17].

For the homogeneous version of the process, much higher acid selectivity values (up to 85%) were achieved at 220 °C and 48 MPa on the carbonyl bimetallic catalyst, Ru–Co–I, formed in situ in molten Bu_4_PBr [18]. A two-stage process scheme via the carbonylation of intermediate methanol is assumed and can be maintained by carefully adjusting the I quantity. The key active complexes are assumed to be [Ru(CO)_3_I_2_]^−^ and [Co(CO)_4_]^−^. The target selectivity rapidly decreases if the Co/Ru ratio drops below 1 and the formation of alcohols with a chain length of 1–3 C atoms becomes predominant, even with iodine-free precursors [19].

Today, studies on the direct production of acetic acid from synthesis gas have been abandoned. However, requirements of modern green chemistry to minimize the number of stages in industrial chemical processes (as well as the growing opportunities of modern catalytic design), have led to renewed attention to the processes. Moreover, research in this area has potential for obtaining important results for the theoretical understanding of synthesis gas transformations on homogeneous catalysts—in this case, to the peculiarities of the formation of species containing two oxygen atoms (monocarboxylic acids, their esters, and diols) in general.

**Figure 1:**
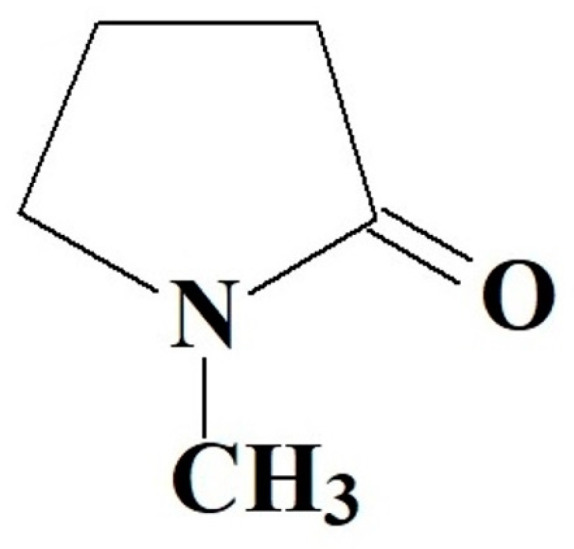
N-methyl-2-pyrrolidone structural formula.

In the present investigation, a homogeneous acetic-acid-synthesis-type catalyst (a carbonyl bimetallic complex, Ru–Co) was prepared in situ from a chlorine-containing precursor using a Li modifier and a solvent, N-methyl-2-pyrrolidone (Figure 1), not previously explored in this field of catalysis. The results of the functionality of this catalytic system at relatively low pressures (10–20 MPa) and moderate temperature (200 °C) are discussed, as well as hypotheses regarding the sequence of oxygenate and hydrocarbon formation explaining its observed composition.

## 2. Results

**Figure 2:**
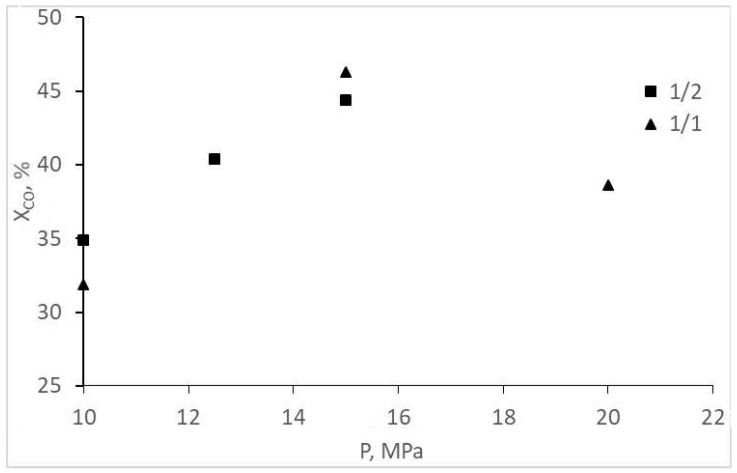
Dependence of CO conversion on the pressure and composition of the synthesis gas.

**Figure 3:**
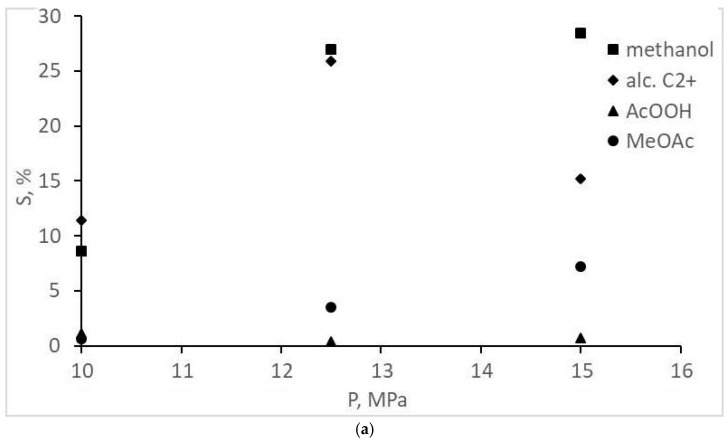

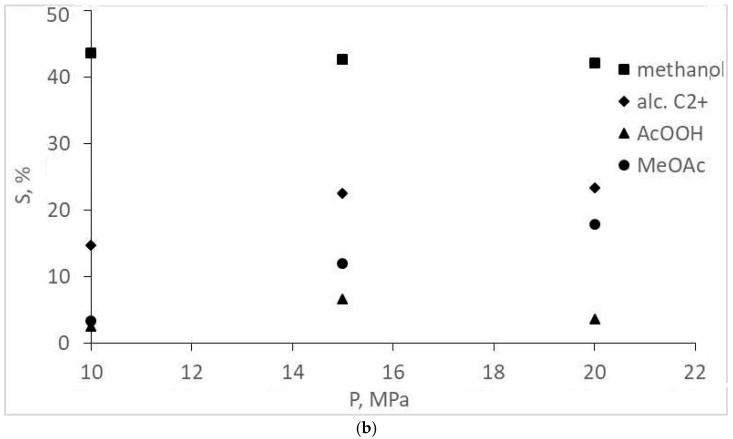
Dependence of oxygenate selectivity on the pressure and composition of the synthesis gas: (**a**) 1CO:2H_2_; (**b**) 1CO:1H_2_.

**Figure 4:**
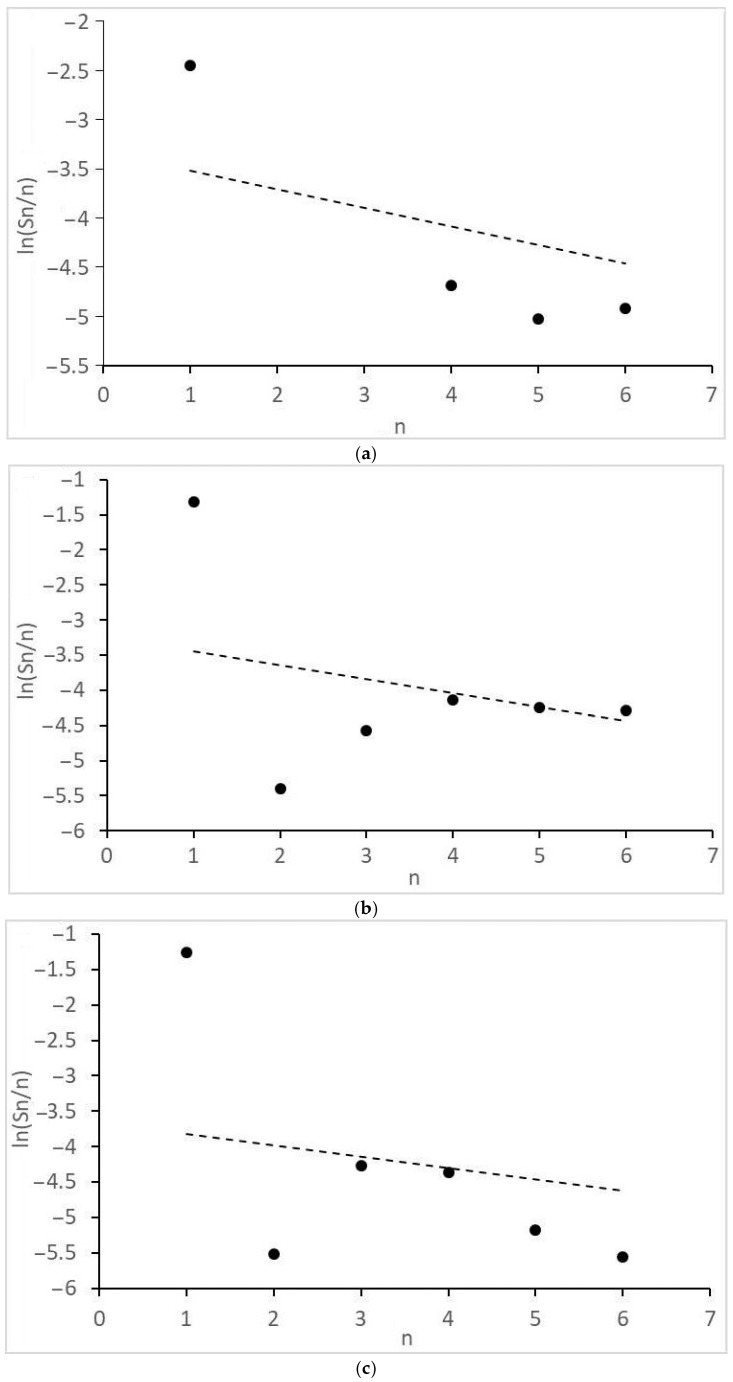

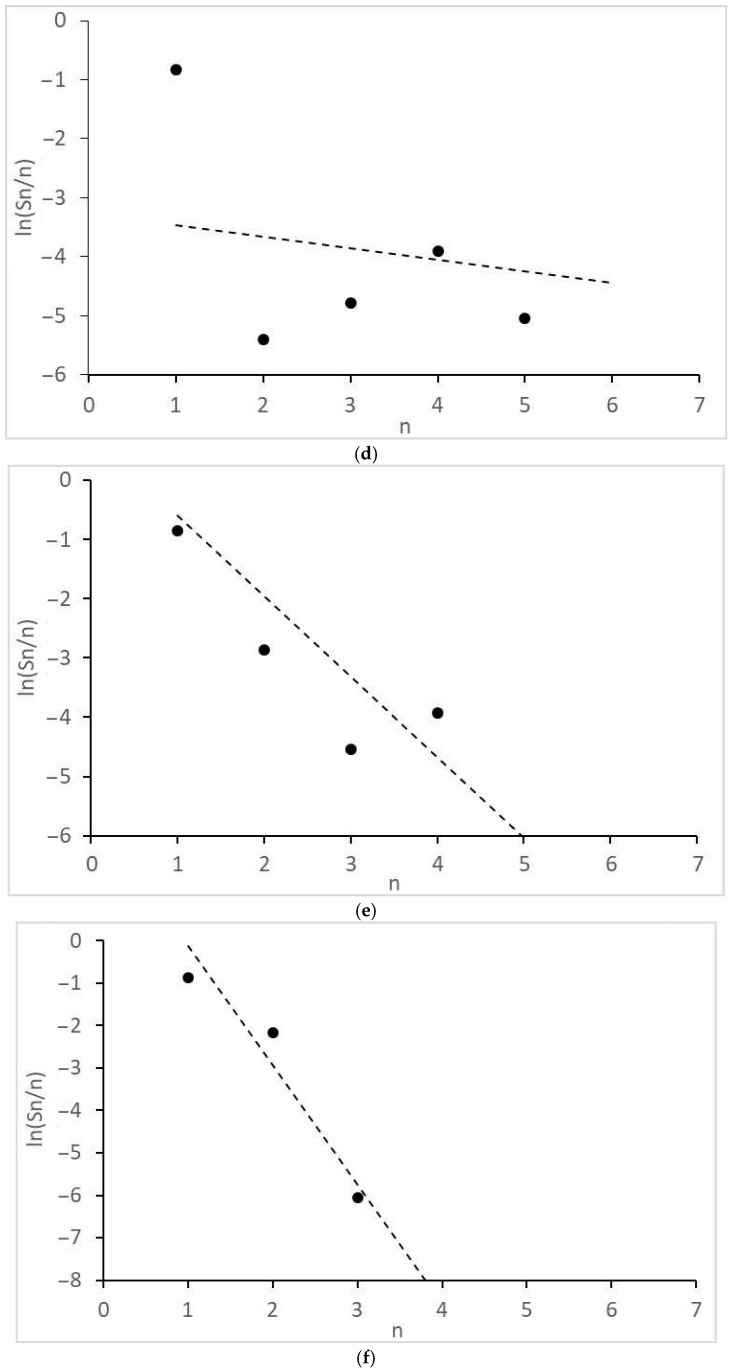
Molecular mass distribution of alcohols: (**a**) 1CO:2H_2_, 10 MPa; (**b**) 1CO:2H_2_, 12.5 MPa; (**c**) 1CO:2H_2_, 15 MPa; (**d**) 1CO:1H_2_, 10 MPa; (**e**) 1CO:1H_2_, 15 MPa; (**f**) 1CO:1H_2_, 20 MPa. n, number of C atoms per product; dot lines, Anderson-Schultz-Flory distribution with optimal α values.

**Figure 5:**
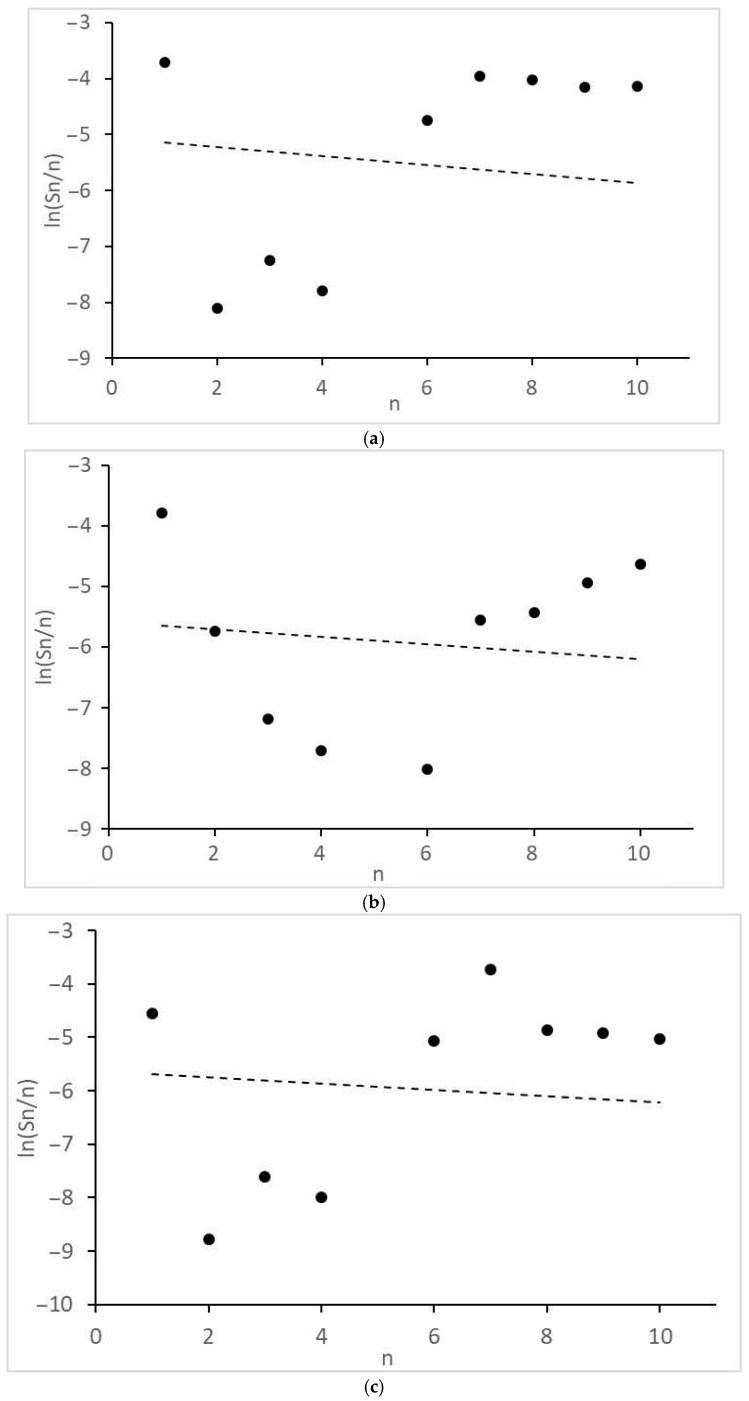

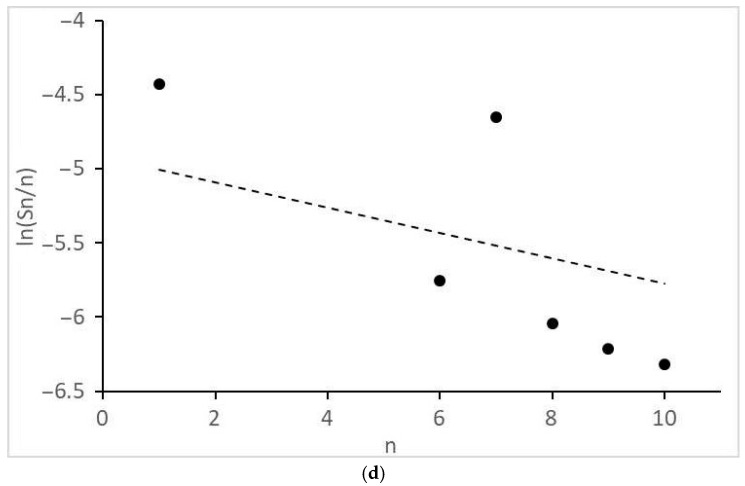
Molecular mass distribution of alkanes: (**a**) 1CO:2H_2_, 10 MPa; (**b**) 1CO:2H_2_, 12.5 MPa; (**c**) 1CO:2H_2_, 15 MPa; (**d**) 1CO:1H_2_, 10 MPa. n, number of C atoms per product; dot lines, Anderson-Schultz-Flory distribution with optimal α values.

The experimental CO conversion and selectivity values for gaseous and liquid products are represented in Table 1 and Figure 2, Figure 3, Figure 4 and Figure 5. The overall CO conversion degree was not high (30–45%) at turnover frequency (TOF) values of 0.005–0.01 s^−1^. The conversion increased significantly with a pressure increase in a particular range, but the increase changed with a pressure increase up to 20 MPa (Figure 2). The target product—acetic acid—was formed in insignificant amounts (selectivity of 7% or less), inferior to methanol and methyl acetate (Table 1, Figure 3). However, an increase in the initial gas CO content to the stoichiometric ratio (1CO:1H_2_) favored the formation of acetic acid, and methanol selectivity increased. The origins of these changes are unclear; they are probably the same ones that cause a decrease in CO conversion at 20 MPa (indirect evidence of the statement is the symbatic changes of these two values with increasing pressure).

Attention should be paid to the absence of acetaldehyde and any esters other than methyl acetate for reactions using 1CO:2H_2_ synthesis gas. At a ratio of 1CO:2H_2_ and a pressure >15 MPa, small amounts of ethyl acetate were also formed (Table 1); acetaldehyde was still not observed.

Of interest are the peculiarities of the formation of acetic acid and other products with two O atoms and the molecular mass distributions of the formed monoatomic alcohols (Figure 4) and alkanes (Figure 5). They revealed significant deviations from the theoretical semi-logarithmic linear distribution (Anderson-Schultz-Flory) for the consecutive carbon chain growth within a homologous series of relevant products. For alcohols, in most cases, methanol (excess), ethanol, and propanol (lack, for propanol—in the little extent) do not obey the distribution; the local experimental distribution maximum was n = 4. For hydrocarbon distributions, in most cases, a sharp selectivity decrease was detected for n = 2–5; a local maximum was observed at n = 7. However, at ≥15 MPa, at H_2_:CO = 1:1, the distribution pattern changed dramatically. For both product series, the chain growth tendency radically decreased. Alcohols longer than C_3_–C_4_ and alkanes above methane and ethane were not detected. Under these conditions, ethyl acetate appeared in the products and acetic acid formation sharply increased.

**Figure 6:**
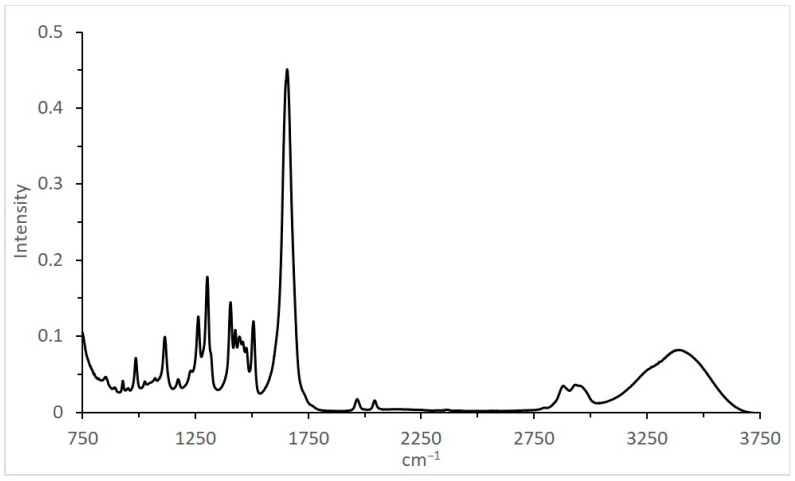
IR spectrum of Ru–Co–Li/N-methylpyrrolidone after synthesis (20 MPa, 1CO:1H_2_).

The IR spectrum for the catalytic system used, at 20 MPa, 1CO:1H_2_ (Table 2, Figure 6), revealed several pronounced peaks in the range of 1000–1700 cm^−1^ related to the solvent and two relatively small and almost identical peaks at 1967 and 2044 cm^−1^, characterizing CO absorption frequencies for carbonyl groups.

## 3. Discussion

### 3.1. Features of Oxygenate Formation

The experimental results reveal significant discrepancies with previous results for the homogeneous synthesis of AcOH and other 2O-containing compounds [18,19]. Whereas the turnover frequencies (TOFs) for the investigated catalyst and its analogs had the same order of magnitude (0.005–0.01 s^−1^ and 0.002–0.005 s^−1^ [18], respectively), the selectivity differed dramatically (Table 3); this is consistent with the different nature of the catalyst components used and the significantly lower pressure used in this study. No notable excess methanol or methyl acetate selectivity cases predominating over AcOH selectivity have been described [18,19] for Co/Ru = 1–2. Moreover, the selectivity predominance of ethyl acetate over methyl acetate was not observed in this work, whereas it was detected repeatedly in other studies [18,19].

The selected composition of the catalyst and its solvent, in general, is novel for homogeneous catalytic CO reactions. In the contemporary trends in the carbonylation of various organic compounds [20,21], the combination of Ru, Co, and Li (regardless of the specifics of the precursors containing these elements) has never been used; the use of the solvent N-methylpyrrolidone for these purposes is also unknown. A catalyst of the composition Ru(PPh_3_)_3_Cl_2_–CoI_2_–LiI is known for the conversion of dimethyl ether into ethanol under the action of CO_2_ and H_2_ [22]; however, the solvent used for this reaction (1,3-dimethyl-2-imidazolidinone) is significantly different from the one used in this work. Lithium promoter and N-methylpyrrolidone were also not used in the above-mentioned investigations [18,19] of direct CO and H_2_ conversion.

Further, all observed alcohol product distributions demonstrated a local methanol excess (Figure 4a–d) or its compliance with the ideal Anderson-Schultz-Flory distribution. This can be considered as indirect—but strong—evidence that methanol formed from the synthesis gas. The equation:

CO + 2H_2_ → CH_3_OH (1)

is not consumed preferably in any of the following sequential reactions: carbonylation into acetic acid:

CH_3_OH + CO → CH_3_COOH (2)

hydroformylation into acetaldehyde:

CH_3_OH + CO + H_2_ → CH_3_CHO + H_2_O (3)

or homologation into methyl acetate:

2CH_3_OH + CO → CH_3_COOCH_3_ + H_2_O (4)

because, if such a consumption had predominance, one could expect a lack of methanol compared with the ideal distribution, not an excess.

Instead of (2)–(4), one can assume for the investigated catalyst the existence of a set of independent direct routes of CO/H_2_ conversion into the corresponding products:

2CO + 2H_2_ → CH_3_COOH (5)

2CO + 3H_2_ → CH_3_CHO + H_2_O (6)

3CO + 4H_2_ → CH_3_COOCH_3_ + H_2_O (7)

No acetaldehyde was detected in this study. Therefore, (6) is not possible for the investigated catalyst and conditions. This agrees with [18,19] for RuCo–Bu_4_PBr; however, no discussion of the acetaldehyde absence for Ru–Co-catalyzed direct syngas transformation took place there, and no comparative analysis of homogeneous and heterogeneous (CH_3_CHO is a typical product for Rh-containing catalysts [14,15,16,17]) cases of acetic acid synthesis exist. Whether the aldehyde is completely (and selectively) hydrogenated without the complete hydrogenation of acetic acid or/and alcohols remains in question.

**Figure 7:**
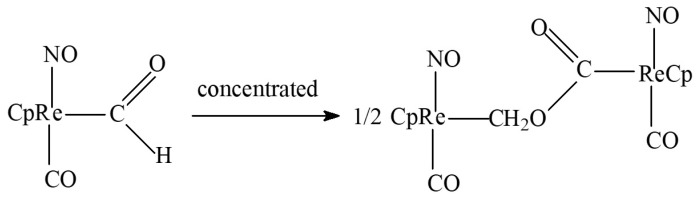
Scheme of probable methyl acetate precursor formation; CpFe = cyclopentadienyl iron dicarbonyl.

The direct formation of methyl acetate (7) is also a questionable process. The only potential precursor of this substance that appears during the homogeneous process without alcohol is the product of the condensation of two formyl ligands (Figure 7 [23,24]). Here, a different catalyst (CpRe) and a specific hydride agent (NaBH_3_CN or HBF_4_-OEt_2_) are required without guaranteeing proper acetate elimination. On the other hand, the methanol homologation into methyl acetate (alone or within a system of other reactions) also requires different catalytic media/ligands (MeI for Co–PPh_3_ [25] or Bu_4_PBr for Co–Ru [19]). Moreover, no acetic acid esterification for the investigated system is to be expected (although this could provide a partial explanation for its low selectivity); this reaction is catalyzed by strong acids, such as H_2_SO_4_, at <100 °C [26,27].

Finally, one can see that, when no ethyl acetate was detected (1CO:2H_2_), the tested catalyst caused a strongly non-ideal distribution of alcohols (Figure 4a–d) with a sharp lack of ethanol. When ethyl acetate was detected (1CO:1H_2_ and >15 MPa), a much more uniform alcohol distribution was observed (Figure 4e,f). Therefore, it is a valid hypothesis that there is no synthesis route during which ethanol is consumed and ethyl acetate is formed.. Then, one can see that, at the transition from 1CO:2H_2_ to 1CO:1H_2_, when ethyl acetate appears, methyl acetate and methanol selectivity strongly increase (Table 1). Hence, one can consider that the homologation reactions:

2CH_3_OH + 2CO + 2H_2_ → CH_3_COOC_2_H_5_ + 2H_2_O (8)

CH_3_COOCH_3_ + CO + 2H_2_ → CH_3_COOC_2_H_5_ + H_2_O, (9)

also barely took place. Due to these considerations, a direct formation of ethyl acetate from the synthesis gas looks like a plausible possibility.

### 3.2. Features of Formation of Long-Chain Alkanes and Alcohols 

The side process catalyzed by Ru–Co–Li/N-methylpyrrolidone is a well-known Fischer-Tropsch synthesis of long-chain (for this case, it is appropriate to say “middle-chain”) aliphatic hydrocarbons. As stated above, no known homogeneous catalyst can provide a full-cycle transformation of CO and H_2_ into hydrocarbon products (one of the latest examples is [28]: a hybrid mixture of homogeneous RuCl_3_ and heterogeneous Ru^0^ catalysts must be used for the synthesis of C_5+_ hydrocarbons via CO_2_ hydrogenation). Here, at H_2_:CO = 2:1 and 10 MPa, C_6_–C_10_ alkanes became the prevailing product (total selectivity 63%, see Table 1). When the pressure reached ≥15 MPa at H_2_:CO = 1:1, liquid alkanes disappeared but a small amount of ethane was detectable (Table 1). There is evidence of limited hydrocarbon chain growth in the investigated conditions (except for 20 MPa). The significant deviations of the molecular mass product distributions of alkanes and alcohols from the Anderson-Schultz-Flory model were mentioned in the previous section.

**Figure 8:**
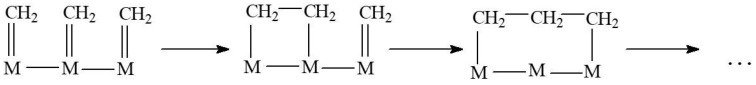
Scheme of hypothetic surface C_1_ condensation (methylene example).

For the Fischer-Tropsch synthesis, the maxima of the product distribution was first discussed as early as the 1970s–1980s [29]. That phenomenon is attributed to the spatial restriction of the carbon chain growth arising from the hypothetical surface condensation of neighboring C_1_ intermediates (Figure 8, case of CH_2_ groups) on a spatially restricted group of active atoms. Therefore, the resulting product distribution shape correlated with the size distribution shape of these groups (presumed Gaussian). The nature of the above-mentioned spatial restriction differs—for heterogeneous Fischer-Tropsch catalysts, that is, the size of micro- or meso-pores of catalyst supports [30,31], especially zeolites with the 3D structure of channels (shape selectivity [32,33,34,35]). This field of Fischer-Tropsch catalysis, however, nowadays, is nearly abandoned (because of the generally low activity of shape-selective systems).

For homogeneous catalysts, the formation of such groups is evident (clusterization and aggregation into nano-particles), and the presence of such clusters/particles in the investigated system has been indirectly confirmed by IR spectroscopy data (Table 2, Figure 6). The combination of CO absorption frequencies (1967 and 2044 cm^−1^) is more relevant for Co_2_(CO)_4_ [36] (1975–2035 cm^−1^, depending on the position and substitution) and even heterogeneous Ru/SiO_2_ [37] (frequencies for bridged 1909–1997 and linear 2035 cm^−1^ modes of adsorbed CO) rather than for mononuclear HCo(CO)_4_ [38] (2050–2100 cm^−1^). The investigated catalyst’s cluster/particle size distribution has not yet been revealed.

Finally, some considerations should be formulated about the distribution model parameters. In order for the product distribution to take place similar to [26], the active atoms, regardless of cluster size, should provide high values of the intrinsic growth factor (*α_int_*→1, see Equation (11a) and (11b) in the next section). For the discussed catalyst, this is roughly manifested in the values of the apparent Anderson-Schultz-Flory coefficient *α_av_*, which is determined using a minimization procedure. For alcohols, *α_av_* ≈ 0.85 and, for hydrocarbons, *α_av_* ≈ 0.93, except for H_2_:CO = 1:1, >15 MPa, where *α_av_* decreases drastically (<0.25). The reasons for the discrepancy between the positions of two product distribution maxima have not yet been discovered; perhaps this is due to the different nature of chain growth active centers for alcohols and alkanes (for example, Ru alone can catalyze only the alcohol chain growth; Co, only the alkanes).

## 4. Materials and Methods

The catalyst was prepared by dissolving precursors (0.026 g of RuCl_3_, TU 2625-066-00196533-2002, BVB-Alliance, Russia; 0.022 g of CoCl_2_, purity > 98%, Lenreactive, Russia; 0.079 g of lithium chloride, purity > 99%, Lenreactive, Russia) in 5 mL of N-methylpyrrolidone (purity > 99%, ECOS-1, Russia). After pouring the prepared solution into a batch-stirring stainless steel reactor (35 mL), a synthesis gas was injected (1CO:1-2H_2_; purity > 99%, PTK Cryogen, Russia; H_2_, Marc 5.0, KM Research Institute, Russia) with a 5 vol% N_2_ admixture (purity > 99%, PTK Cryogen, Russia) up to operating pressure (10.0–20.0 MPa). After that, the reactor was heated to 200 °C with constant stirring and kept for 4 h; then, the reactor was cooled to room temperature, and sampling for analysis was conducted.

The analysis of the gas samples was carried out using a gas chromatograph “Chromos GH-1000” (CHROMOS Engineering, Dzerzhinsk) with a catarometer. To determine CO, CH_4_, and N_2_ quantities, separation was carried out on a molecular sieve NaX/3X (2 m × 3 mm) under isothermal conditions (50 °C) in a 20 mL/min He carrier (Brand 5.0, KM Research Institute, Russia). To determine CO_2_ and C_1_–C_4_ hydrocarbon quantities, separation was carried out on HayeSep R adsorbent (2 m × 3 mm) under thermo-programmable conditions (50–200 °C, 8 °C/min) in a 20 mL/min He carrier. The H_2_ content was calculated according to the mass balance of the gas inlet/outlet.

The liquid reaction media analysis was conducted using a Thermo Focus DSQII CM spectrometer (Varian VF-5ms capillary column 15 m × 0.25 mm, eluent layer thickness 0.25 μm, injector temperature 270 °C, He) under thermo-programmable conditions (40–300 °C, increase of 15 °C/min, 300 °C maintained for 10 min). Mass spectrometer operating mode: ionization energy 70 eV, source temperature 230 °C, scanning range 10–800 Da at 2 scans/s, single resolution over the entire mass range.

IR spectra were recorded via reflection using a HYPERION-2000 IR spectrometer coupled to an IFS-66ν/s Bruker IR Fourier transducer (600–4000 cm^–1^).

The TOF (s^−1^) of the catalyst was calculated under the assumption that the active center of the catalyst is a pair of two atoms, Ru + Co:

(10) $$TOF=\frac{P}{0.101}\frac{V_{r}X_{\mathrm{CO}}}{22.4\left(H_{2}/\mathrm{CO}+1\right)}/\left(\frac{m_{{\mathrm{RuCl}}_{3}}}{M_{{\mathrm{RuCl}}_{3}}}+\frac{m_{{\mathrm{CoCl}}_{3}}}{M_{{\mathrm{CoCl}}_{2}}}\right)/2\;$$

where *X*_CO_ is CO conversion, *V_r_* is reaction zone volume (0.03 L), *P* is initial pressure (MPa), *m*_RuCl_3_;CoCl_2__ is the amount of RuCl_3_ or CoCl_2_ (g), *M*_RuCl_3__ = 207.5 g/mol, and *M*_CoCl_2__ = 130 g/mol.

Theoretical mass distributions of different kinds of synthesis products were calculated according to the Anderson-Schultz-Flory equation [39]:

(11a) $$m_{n}/n={\left(1-\alpha \right)}^{2}\alpha^{n-1}$$

(11b) $$\ln\left(m_{n}/n\right)=\ln\left({\left(1-\alpha \right)}^{2}/\alpha \right)+n\ln\alpha$$

where *m_n_* is the fraction of the initial CO converted into a product with *n* carbon atoms and 0 < *α* < 1 is the chain growth probability presumed independent of *n*. If an experimental distribution deviates from the linear form, then the constant chain growth probability is not suitable.

Optimal *α_av_* values for (10) were determined using the method of least squares (calculations were carried out using Excel 2013) for differences between theoretical and experimental ln(*m_n_*/*n*) values (see Figure 4 and Figure 5).

## 5. Conclusions

The homogeneous synthesis from CO and H_2_ over a Co–Ru–Li/N-methylpyrrolidone catalyst at moderately high pressures (10.0–20.0 MPa)—which seems to have been studied repeatedly—turns out to be new and original, not only concerning the composition of the catalyst. Despite the unreached selective production of acetic acid (assumed at the beginning of this study), the experiments made it possible to reveal several interesting and unusual phenomena and consider some hypotheses about their origins:

(a) Combinations of selectivity values of methanol, ethanol, methyl and ethyl acetate, and acetic acid, and the complete absence of acetaldehyde, can be interpreted as an indication of the formation of both acetates and acetic acid by direct independent CO and H_2_ conversion without any alcohol formation. This is very unusual for a homogeneous catalytic synthesis of two-oxygen-containing substances.
(b) Significant and different deviations of the molecular mass distributions of alkanes and alcohols from the Anderson-Schultz-Flory model, characterized by local maxima and minima, are observed. These tendencies may indicate significant restrictive control of the growth of carbon chains.

The origins of such a behavior related closely with in situ transformations of the studied catalytic system: interactions of chlorine-containing precursor and lithium modifier and the solvent, size, and composition of Ru and Co (both mono- and bi-metallic) clusters with different numbers of active centers for assumed C_1_ condensation (such as, for example, Figure 7). Again, we highlight that N-methylpyrrolidone has never been studied for synthesis gas transformations. The catalyst considered homogeneous, therefore, may turn out to be a prototype for original homogeneous or nanocluster catalysts for selective transformations of a synthesis gas into long-chain products of different classes—from hydrocarbons to carboxylic acids.

## Figures and Tables

**Table 1:** Pressure and CO:H_2_ ratio dependencies of the CO conversion and product selectivity.

Initial CO:H_2_	1/2	1/2	1/2	1/1	1/1	1/1
Initial pressure, MPa	10.0	12.5	15.0	10.0	15.0	20.0
CO conversion, %	34.9	40.4	44.4	31.9	46.3	38.6
TOF, s^−1^ (Equation (10))	0.005	0.008	0.010	0.007	0.002	0.002
Selectivity, %						
CO_2_	12.5	17.2	7.3	20.5	12.5	10.1
CH_4_	2.5	2.3	1.1	1.2	0.74	0.31
Ethane	0.031	0.32	0.016	0.0	0.026	0.0
Propane	0.072	0.075	0.055	0.0	0.0	0.0
Butanes	0.042	0.045	0.035	0.0	0.0	0.0
Hexanes	5.2	0.18	3.8	1.9	0.0	0.0
Heptanes	13.5	2.7	16.8	6.7	0.0	0.4
Octanes	14.3	3.5	6.2	1.9	0.0	0.0
Nonanes	14.2	6.5	6.6	1.8	0.0	0.0
Decanes	15.9	9.7	6.5	1.8	0.0	0.0
Methanol	8.6	27.0	28.5	43.7	42.7	42.2
Ethanol	0.0	0.88	0.76	0.93	11.4	22.7
Propanol	0.0	3.1	4.2	2.5	3.2	0.69
Butanol+iso-propylmethylketone	3.7	6.4	5.1	8.1	7.9	0.0
Pentanol	3.3	7.2	2.8	3.2	0.0	0.0
Hexanol	4.4	8.3	2.3	0.0	0.0	0.0
Acetic acid	1.1	0.43	0.71	2.5	6.6	3.6
Methyl acetate	0.64	3.5	7.2	3.3	12.0	17.9
Ethyl acetate	0.0	0.0	0.0	0.0	2.9	2.1

**Table 2:** Peak intensity of IR spectrum of Ru–Co–Li/N-methylpyrrolidone after synthesis (20 MPa, 1CO:1H_2_). Bold type—CO absorption frequencies for hypothetical carbonyl clusters.

cm^−1^	Intensity
3399	0.0818
2951	0.0351
2879	0.0348
**2044**	**0.0156**
**1967**	**0.0173**
1656	0.451
1506	0.120
1475	0.0848
1460	0.0929
1445	0.0995
1427	0.108
1405	0.144
1302	0.178
1262	0.126
1229	0.0548
1173	0.0436
1114	0.0992
1071	0.0449
1026	0.0406
985	0.0715
928	0.0415
851	0.0467
802	0.0507

**Table 3:** Comparison of selectivity of homogeneous Co–Ru-containing catalysts for synthesizing oxygen-containing compounds.

Catalyst	This Study		Ru_3_(CO)_12_/CoI_2_/ Bu_4_PBr [18] *		Ru_3_(CO)_12_/ Co_2_(CO), Bu_4_PBr [19] *	
T, °C	200	200	200	200	220	220
CO:H_2_	1/2	1/1	1/1	1/1	1/1	1/1
Pressure, MПa	15.0	15.0	48.0	48.0	55.0	55.0
Co/Ru	1.25	1.25	1	1.5	1	2
Selectivity, % *						
Methanol	28.5	42.7	2	~0	25	8
Ethanol	0.76	11.4	~0	~0	21	8
Acetic acid	0.71	6.6	64	85	26	56
Methyl acetate	7.2	12.0	3	15	10	10
Ethyl acetate	0.0	2.9	10	~0	13	16
CH4	1.1	0.74	20	~0	~0	~0

* without CO_2_.

## Data Availability

Not applicable.
